# Antimicrobial Activity of Phytic Acid: An Emerging Agent in Endodontics

**DOI:** 10.3389/fcimb.2021.753649

**Published:** 2021-10-26

**Authors:** Rania Nassar, Mohannad Nassar, Morgana E. Vianna, Nerissa Naidoo, Fatma Alqutami, Eleftherios G. Kaklamanos, Abiola Senok, David Williams

**Affiliations:** ^1^College of Medicine, Mohammed Bin Rashid University of Medicine and Health Sciences, Dubai, United Arab Emirates; ^2^Oral and Biomedical Sciences, School of Dentistry, College of Biomedical and Life Sciences, Cardiff University, Cardiff, United Kingdom; ^3^Department of Preventive and Restorative Dentistry, College of Dental Medicine, University of Sharjah, Sharjah, United Arab Emirates; ^4^Hamdan Bin Mohammed College of Dental Medicine, Mohammed Bin Rashid University of Medicine and Health Sciences, Dubai, United Arab Emirates

**Keywords:** antibacterial, antibiofilm activity, EDTA, dental biofilm, *Enteroccoccus faecalis*, phytic acid

## Abstract

**Background:**

Phytic acid (IP6) is a promising and emerging agent, and because of its unique structure and distinctive properties, it lends itself to several applications in dentistry. Recently, IP6 was proposed as a potential chelating agent in endodontics. However, there is limited knowledge regarding its antimicrobial and antibiofilm effectiveness. The aims of this study, were therefore to evaluate the antimicrobial and antibiofilm activities of IP6 against a range of microbial species and compare these with ethylenediaminetetraacetic acid (EDTA) and sodium hypochlorite (NaOCl). The contact time required for IP6 to exert its bactericidal effect on *Enterococcus faecalis* was also determined.

**Methods:**

The inhibitory and biocidal activities of IP6, EDTA and NaOCl were assessed using a broth microdilution assay against 11 clinical and reference strains of bacteria and a reference strain of *Candida albicans*. The contact time required for various IP6 concentrations to eliminate planktonic cultures of *E. faecalis* was determined using a membrane filtration method according to BS-EN-1040:2005. IP6 bactericidal activity was also evaluated using fluorescent microscopy, and the antibiofilm activity of the test agents was also determined.

**Results:**

IP6 was biocidal against all tested microorganisms. At concentrations of 0.5%, 1% and 2%, IP6 required 5 min to exert a bactericidal effect on *E. faecalis*, while 5% IP6 was bactericidal after 30 s. IP6 also eradicated biofilms of the tested microorganisms. In conclusion, IP6 had notable antimicrobial effects on planktonic and biofilm cultures and exhibited rapid bactericidal effects on *E. faecalis*. This research highlighted, for the first time the antimicrobial and antibiofilm properties of IP6, which could be exploited, not only in dental applications, but also other fields where novel strategies to counter antimicrobial resistance are required.

## 1 Introduction

Microbial biofilms are integral to the failure of root canal treated teeth (Ricucci et al., 2016; Neelakantan et al., 2017; Pereira et al., 2017). Key objective of root canal treatment is therefore the reduction or elimination of biofilm from the root canal system through use of antimicrobial agents (Rahimi et al., 2014; Jhajharia et al., 2015). Ideally, root canal irrigants should have broad antibacterial efficacy, be able to remove the smear layer, dissolve organic tissue and should not be toxic to surrounding tissue (Haapasalo et al., 2014). Sodium hypochlorite (NaOCl) and ethylenediaminetetraacetic acid (EDTA) are the main irrigants currently used in endodontics (Cheung and Stock, 1993; Hülsmann et al., 2003; Haapasalo et al., 2014). NaOCl exhibits bactericidal activity (Retamozo et al., 2010) and can dissolve vital and necrotic organic tissue (Naenni et al., 2004). However, NaOCl has no effect on the inorganic components of the smear layer that forms following mechanical instrumentation (Torabinejad et al., 2003a). As a result, a metal chelator is required as an adjuvant irrigating solution to fully remove the smear layer. Due to its ability to remove the smear layer, EDTA is the chelating agent of choice for this purpose (Violich and Chandler, 2010). However, EDTA has several drawbacks, including toxicity to periradicular tissue and an inability to eradicate bacteria which may have a negative impact on treatment outcome (Spangberg et al., 1973; Tanomaru Filho et al., 2002; Amaral et al., 2007). In addition, EDTA is considered a pollutant (Sillanpää, 1997; Oviedo and Rodríguez, 2003), and the relatively high EDTA concentration (15-17%) used in dentistry (Haapasalo et al., 2014) is a concern. Therefore, it is imperative to identify alternative chelating agents for root canal treatment.

Phytic acid (IP6) is the major storage form of phosphorus in plant seeds and grain and is extracted using a simple and cost effective process (Bloot et al., 2021). Due to its unique structure and distinctive properties, IP6 lends itself to several dental applications (Nassar et al., 2021), such as dental adhesives (Nassar et al., 2013; Forgione et al., 2021) and an endodontic irrigant (Nassar et al., 2015). The high negative charge density of IP6 facilitates chelating activity with cations, thus IP6 has been proposed as an alternative root canal chelating agent with an ability to remove the smear layer, whilst also being biocompatible with osteoblast-like cells (Nassar et al., 2015). *Enterococcus faecalis* is one of the most frequently encountered microorganisms in cases of endodontic treatment failure (Rôças et al., 2004), where persistent infection is commonplace and possibly related to the ability of this species to form biofilms, invade dentinal tubules and resist bactericidal agents in endodontic procedures (Zhang et al., 2015; Alghamdi and Shakir, 2020). *Streptococcus* and S*taphylococcus* species are also often isolated from persistent root infections (Pinheiro et al., 2003; Murad et al., 2014). In addition to bacteria, fungi may also be involved in root canal infections and in such cases, *Candida albicans* is the species most frequently isolated (Persoon et al., 2017). In preliminary *in vitro* experiments, we reported that IP6 had bactericidal effects on planktonic *E. faecalis* (Nassar and Nassar, 2017). However, there is a paucity of knowledge on the antimicrobial spectrum of IP6 and its mechanism of action. There is therefore a need to study IP6's antimicrobial activity against a range of Gram-positive and Gram-negative bacteria with different susceptibility profiles. As root canal infections are biofilm-associated (Ricucci and Siqueira, 2010), it is important that the irrigant eradicates biofilm and constituent bacteria. To the best of our knowledge, there are no reports of IP6’s antibiofilm activity, nor the contact time and concentration required for antimicrobial effects against *E. faecalis*. This study was therefore undertaken to investigate the antimicrobial and antibiofilm spectrum of IP6 and compare this with EDTA and NaOCl. In addition, the contact time required for different concentrations of IP6 to its exert bactericidal effects on *E. faecalis* was also determined.

## 2 Material and Methods

### 2.1. Experimental Agents and Test Microorganisms

Test agents included IP6 (50% (w/w) in H_2_O) (Sigma Aldrich), 5.25% NaOCl (Chlorax; Cerkamed, Poland) and EDTA (0.5 M, 18.61% (w/v), pH 8) (Sigma Aldrich). Two-fold serial dilutions (up to 512-fold dilution) of these test agents were prepared for susceptibility testing and the highest IP6 concentration was 40%. Susceptibility of both planktonic and biofilm cultures of reference and clinical microorganisms to the agents was undertaken. Reference strains were commercially sourced, while clinical strains were obtained from stock cultures at the School of Dentistry, Cardiff University (**Table 1**).

**Table 1:** List of the reference and clinical microorganisms used in this study.

Microorganism	Stain reference/origin
*Enterococcus faecium* (VRE)	Clinical strain (Kuriyama et al., 2003)
*Enterococcus faecalis* (VSE)	Oral strain (United Kingdom) (Lins et al., 2019)
*Enterococcus faecalis* (VRE)	Hospital/Environnemental strain (Kuriyama et al., 2003)
*Enterococcus faecalis* (VSE)	Clinical strain (Japan) (Lins et al., 2019)
*Staphylococcus aureus* (MSSA)	ATCC 25923
*Staphylococcus aureus* (MRSA)	ATCC 43300
*Pseudomonas aeruginosa*	ATTCC 9027
*Escherichia coli*	ATCC 25922
*Enterococcus faecalis* (VSE)	ATCC 29212
*Enterococcus faecalis* (VRE)	ATCC 51299
*Streptococcus mutans*	ATCC 25175
*Candida albicans*	ATCC 90028

VSE, Vancomycin Susceptible Enterococcus; VRE, Vancomycin Resistant Enterococcus; MRSA, Methicillin Resistant Staphylococcus aureus; MSSA, Methicillin Susceptible Staphylococcus aureus.

### 2.2. Antimicrobial Activity Against Planktonic Cultures

#### 2.2.1. Minimum Inhibitory Concentration (MIC) of Test Agents

The antimicrobial activities of IP6, EDTA and NaOCl were assessed against planktonic cultures using a broth microdilution assay to determine minimum inhibitory concentrations (MICs). Bacteria were cultured on Tryptone Soya Agar (TSA) for 24 h at 37°C, while *C. albicans* was cultured on Sabouraud Dextrose Agar (SDA) in 5% CO_2_, at 37°C for 48 h. Isolated colonies were used to prepare bacterial suspensions in Mueller Hinton Broth (MHB), whilst *C. albicans* suspensions were prepared in RPMI-1640 medium (Lonza, USA). Microbial suspensions were adjusted to a turbidity equivalent to a 0.5 McFarland standard, and then diluted 100-fold in either MHB (for bacteria) or RPMI-1640 (for *C. albicans*). One hundred μl of each suspension was then added to appropriate U-shaped wells of a 96-well microtiter plate containing 100 μl of diluted test antimicrobial. Controls included microorganisms incubated in culture medium devoid of antimicrobial, or uninoculated MHB and RPMI-1640 media. Microtiter plates were incubated at 37°C for 24 h. The MIC was defined as the lowest antimicrobial concentration required to inhibit visible growth after 24 h incubation. Three independent experiments, each including 3 replicates were performed.

#### 2.2.2. Minimum Biocidal Concentration of Test Agents

The minimum biocidal concentration (MBC) was determined by subculturing the contents of the appropriate microtiter plate wells (after MIC testing) into antimicrobial-free medium and assessing subsequent growth. Briefly, microtiter plate contents were added to 2 ml of sterile phosphate-buffered saline (PBS) and centrifuged at 6000 rev/min (Eppendorf 5424) for 10 min. The supernatant was discarded, and the pellet resuspended in 2 ml of Tryptone Soya Broth (TSB) or RPMI-1640 depending on the organism. Twenty μl of the suspension was then cultured in triplicate on TSA. Agar plates and suspensions were incubated for 24-72 h at 37°C and examined for colony presence and turbidity, respectively. The MBC was the lowest antimicrobial concentration that resulted in no microbial growth. Three independent experiments, each including 3 replicates were performed.

#### 2.2.3. Fluorescent Microscopy

Bacterial viability was evaluated using the Live/Dead^®^ BacLight™ bacterial viability kit (Invitrogen Ltd., UK). A bacterial suspension of *E. faecalis* (oral strain) (Lins et al., 2019) with a turbidity equivalent to a 0.5 McFarland standard was prepared. The bacterial suspension was treated with an equal volume of either IP6 at the MBC concentration, or sterile distilled water, and incubated at 37°C. A 500-µl volume of the mixture was removed at different time points (30 min, 1 h, and 2 h) and centrifuged at 6000 rev/min (Eppendorf 5424) for 10 min. The pellet was then resuspended in 200 µl of the viability dye mixture prepared in PBS and incubated for 15 min in the dark at room temperature. Samples were then examined using an Olympus BX63 automated fluorescent microscope.

#### 2.2.4. Determination of IP6 Bactericidal Contact Time

*E. faecalis* ATCC 29212 was used to determine bactericidal contact times. The final concentrations of IP6 tested were 0.5%, 1%, 2%, and 5%. A membrane filtration method was used in accordance with BS-EN-1040:2005 (BSi, 2006) to determine the contact time (30 s, 1 min or 5 min) required to exert bactericidal activity. Isolated colonies of *E. faecalis* on Brain Heart Infusion agar plates were suspended in sterile tryptone sodium chloride solution. The suspension was adjusted to a turbidity equivalent to a 0.5 McFarland standard and the number of total colony forming units (CFUs)/ml was determined by serial dilution and culture using a spread technique. To determine bactericidal activity at a specific contact time, 1 ml of the bacterial suspension was mixed with 1 ml of sterile distilled water. To these mixtures, 8 ml of a test agent was added for 30 s, 1 min, or 5 min. At the end of each contact time, three 0.1-ml samples were immediately filtered and rinsed with 150 ml of sterile distilled water. The filter membrane was then transferred to the surface of a TSA plate. All plates were incubated at 37°C for 24-48 h. Tests were performed on a minimum of three different occasions. Control and validation tests were undertaken throughout the experiment according to BS-EN-1040:2005 (BSi, 2006). Purity checks of the bacterial suspensions and sterility of reagents were achieved by culturing on TSA. The log reduction (R) was calculated using the following equation: logR= logN_0_-logN_a_, where N_0_ was the bacterial count (CFU/ml) in the test mixture at contact time of 0 (before treatment) and N_a_ was the surviving bacterial count (CFU/ml) after the contact time (post treatment). A concentration of IP6 was considered to exert bactericidal activity when it produced a log reduction of 5 or more (BSi, 2006).

### 2.3. Determination of Minimum Biofilm Eradication Concentration of Test Agents

Isolated colonies from 24 h agar cultures of bacteria and 48 h agar cultures of *C. albicans* were used to prepare microbial suspensions in MHB or RPMI-1640, respectively. The resulting suspension was adjusted to a turbidity equivalent to a 0.5 McFarland standard for bacteria and a 1.0 McFarland standard for *C. albicans*. One hundred µl of the microbial suspension was then added to the wells of a 96-well (flat-bottom) microtiter plate. Peripheral wells were unused to avoid potential edge effects. Microtiter plates were incubated for 24 h at 37°C to facilitate biofilm formation. After incubation, culture medium was aspirated, and each well washed twice with 150 µl of PBS to remove planktonic cells and any excess washing medium was aspirated. One hundred µl of MHB or RPMI-1640 was added to each well, together with 100 µl of diluted test agent. Controls included uninoculated wells with fresh culture medium only or untreated biofilms. The plates were incubated for 24 h. Antimicrobial solutions were removed, and the wells gently washed twice with 200 µl of PBS. Two hundred µl of MHB or RPMI-1640 were added to the wells and the plates were incubated for 24-72 h. Microbial growth was then assessed visually and by measuring turbidity at 630 nm using a plate reader. The minimum biofilm eradication concentration (MBEC) was determined relative to the turbidity of a growth control (100% growth) and a medium-only control (0% growth). The MBEC was determined when there was no visual turbidity or the spectrophotometric readings were ≥80% reduced relative to the untreated control. Three independent experiments, each including 3 replicates were performed.

## 3 Results

### 3.1. Minimum Inhibitory Concentration (MIC)

All agents were effective at inhibiting planktonic growth of test microorganisms. MICs for IP6 were 0.156% for all bacteria except for *S. mutans*, which was 0.078%, and 1.25% for *C. albicans*. NaOCl inhibited bacteria and *C. albicans* growth at 0.041% and 0.005%, respectively. While the MICs for EDTA ranged between 0.018% and 0.145% (**Table 2**).

**Table 2:** Minimum inhibitory concentration (MIC) and Minimum bactericidal concentration (MBC) of IP6, EDTA and NaOCl against test microorganisms.

Microorganism	IP6		EDTA		NaOCl	
	MIC (%)	MBC (%)	MIC (%)	MBC (%)	MIC (%)	MBC (%)
*E. faecium* (VRE) Clinical strain (Kuriyama et al., 2003)	0.156	0.313	0.018	R	0.041	0.041
*E. faecalis* (VSE) Oral strain (UK) (Lins et al., 2019)	0.156	0.313	0.036	R	0.041	0.041
*E. faecalis* (VRE) Hospital/Environnemental strain (Kuriyama et al., 2003)	0.156	0.313	0.018	R	0.041	0.041
*E. faecalis* (VSE) (Japan) Clinical strain (Lins et al., 2019)	0.156	0.313	0.018	R	0.041	0.041
*S. aureus* (MSSA) ATTCC 25923	0.156	0.313	0.018	R	0.041	0.041
*S. aureus* (MRSA) ATCC 43300	0.156	0.313	0.018	R	0.041	0.041
*P. aeruginosa* ATCC 9027	0.156	0.156	0.145	9.305	0.041	0.041
*E. coli* ATCC 25922	0.156	0.313	0.072	2.326	0.041	0.041
*E. faecalis* (VSE) ATCC 29212	0.156	0.313	0.072	R	0.041	0.041
*E. faecalis* (VRE) ATCC 51299	0.156	0.313	0.036	R	0.041	0.041
*S. mutans* ATCC 25175	0.078	0.313	0.018	R	0.041	0.041
*C. albicans* ATCC 90028	1.25	10	0.018	R	0.005	0.005

VSE, Vancomycin Susceptible Enterococcus; VRE, Vancomycin Resistant Enterococcus; MRSA, Methicillin Resistant Staphylococcus aureus; MSSA, Methicillin Susceptible Staphylococcus aureus. R denotes that microorganism was resistant to all concentrations tested.

### 3.2. Minimum Bactericidal Concentration (MBC) of Test Agents

**Table 2** shows the MBCs for test agents. IP6 was bactericidal against all bacteria at a concentration of 0.313%, which was generally two-fold higher than the MIC. Exceptions to this were for *P. aeruginosa* where the MBC and MIC were the same, and for *S. mutans*, where the MBC was 4-fold higher than the MIC. IP6 exerted biocidal activity against *C. albicans* at higher concentrations (10%) than the MIC. NaOCl was bactericidal at 0.041%, whilst EDTA was not bactericidal, except for *E. coli* and *P. aeruginosa* at concentrations of 2.326% and 9.305%, respectively.

### 3.3. Fluorescent Microscopy

**Figure 1:**
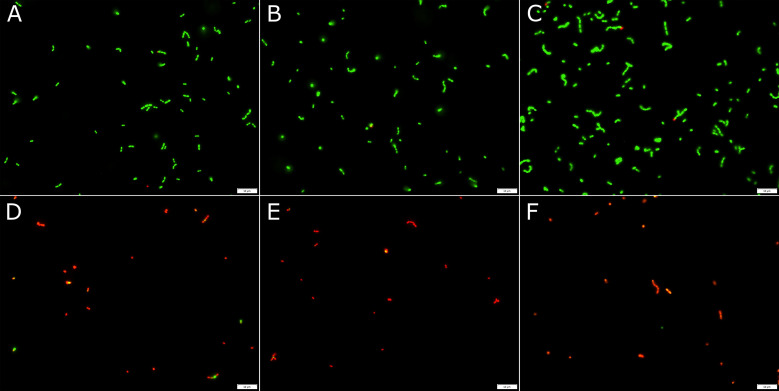
Fluorescent microscopy images of *E. faecalis* treated with either sterile distilled water **(A–C)** for 30 min, 1 h or 2 h, respectively, or 0.313% IP6 **(D–F)** for 30 min, 1 h or 2 h, respectively. Bacteria were stained with the Live/Dead^®^BacLight™ bacterial viability kit. Red staining indicates membrane damage.

Fluorescent microscopy revealed that the number of dead (red stained) *E. faecalis* cells increased with duration of contact time with 0.313% IP6 (**Figure 1**). The emission of red fluorescence from cells stained with propidium iodide (PI) indicated loss of membrane integrity, as PI only penetrates cells with compromised membranes.

### 3.4. Antimicrobial Contact Time

**Table 3** shows the log reduction of *E. faecalis* ATCC 29212 planktonic cultures after IP6 treatment at different contact times. All tested concentrations of IP6 were effective at killing the bacteria, but at different contact times. IP6 (0.5%) required 5 min to exert a bactericidal effect, while at 1% and 2% IP6 exerted a bactericidal effect, with total reduction of viable cells counts at 5 min. IP6 (5%) required 30 s to exert a bactericidal effect and 1 min to give total reduction of viable cells counts.

**Table 3:** Log reduction of *E. faecalis* ATCC 29212 compared to the untreated control (log N0) using IP6 for 30 s, 1 min or 5 min.

		0.5% IP6		1% IP6		2% IP6		5% IP6	
		Log N0	Log R	Log N0	Log R	Log N0	Log R	Log N0	Log R
Contact time	30 s	7.13 (± 0.18)	< 3.91	7.13 (± 0.18)	< 3.91	7.13 (± 0.18)	< 3.91	7.08 (± 0.12)	5.52 (± 0.73)
	1 min	7.13 (± 0.18)	< 3.91	7.13 (± 0.18)	< 3.91	6.99 (± 0.22)	< 3.78	7.23 (± 0.10)	7.23 (± 0.10)
	5 min	7.04 (± 0.10)	5.37 (± 0.43)	7.13 (± 0.18)	7.13 (± 0.18)	7.13 (± 0.18)	7.13 (± 0.18)	7.23 (± 0.10)	7.23 (± 0.10)

Standard Error of Means are shown in parentheses.

### 3.5. Minimum Biofilm Eradication Concentration (MBEC)

IP6 was able to eradicate established biofilms of all tested microorganisms with an MBECs ranged between 0.156% and 10%. The highest MBEC for IP6 was against *C. albicans* (**Table 4**). NaOCl eradicated biofilms of all tested organisms at concentrations between 0.041% and 0.164% (**Table 4**). EDTA did not eradicate biofilms of the tested organisms, except for *E. coli* at concentrations between 2.3% and 9.3% (**Table 4**).

**Table 4:** Minimum biofilm eradication concentration (MBEC) of IP6, EDTA or NaOCl against test microorganisms.

	IP6			EDTA			NaOCl		
	Minimum (%)	Maximum (%)	Mode (%)	Minimum (%)	Maximum (%)	Mode (%)	Minimum (%)	Maximum (%)	Mode (%)
*E. faecium* (VRE) Clinical strain (Kuriyama et al., 2003)	0.313	2.50	1.25		R		0.041	0.082	0.082
*E. faecalis* (VSE) Oral strain (UK) (Lins et al., 2019)	0.625	1.25	0.625		R		0.02	0.082	0.082
*E. faecalis* (VRE) Hospital/Environmental strain (Kuriyama et al., 2003)	0.313	2.50	0.313^a^		R		0.041	0.164	0.041^a^
*E. faecalis* (VSE) Clinical strain (Japan) (Lins et al., 2019)	0.625	2.50	0.625		R		0.082	0.082	0.082
*S. aureus* (MSSA) ATTCC 25923	0.625	2.50	2.50		R		0.082	0.082	0.082
*S. aureus* (MRSA) ATCC 43300	0.625	2.50	0.625		R		0.082	0.082	0.082
*P. aeruginosa* ATCC 9027	0.156	1.25	0.313^a^		R		0.041	0.082	0.082
*E. coli* ATCC 25922	0.156	0.625	0.156^a^	2.326	9.305	2.326	0.082	0.082	0.082
*E. faecalis* (VSE) ATCC 29212	0.313	2.50	0.625		R		0.041	0.082	0.041
*E. faecalis* (VRE) ATCC 51299	0.313	1.25	0.625		R		0.041	0.082	0.082
*S. mutans* ATCC 25175	0.156	0.313	0.313		R		0.041	0.082	0.082
*C. albicans* ATCC 90028	1.25	10	1.25^a^		R		0.041	0.041	0.041

VSE, Vancomycin Susceptible Enterococcus; VRE, Vancomycin Resistant Enterococcus; MRSA, Methicillin Resistant Staphylococcus aureus; MSSA, Methicillin Susceptible Staphylococcus aureus.

a Multiple modes exist. The lowest value is shown. R, resistance to all concentration tested.

## 4 Discussion

An ideal endodontic irrigant should possess several properties including an ability to remove the smear layer, have biocompatibility with vital tissues, and should have a broad spectrum antimicrobial effect with a rapid action to minimize adverse effects on root canal dentin (Haapasalo et al., 2014). However, none of the currently used irrigants fulfill all of these properties (Calt and Serper, 2002).

Due to its broad spectrum antimicrobial activity and ability to dissolve organic tissue (Zehnder et al., 2005), NaOCl is the gold standard disinfectant in endodontic treatment, despite its well-recognised drawbacks (Zehnder, 2006). NaOCl lacks the ability to remove inorganic materials, hence the necessity to use chelating agents following NaOCl treatment (Zehnder, 2006). EDTA is typically used as a chelating agent in root canal treatment to remove the smear layer (Calt and Serper, 2002) with a recommended contact time of 1-5 min. Despite the popularity of EDTA, the associated disadvantages of this irrigant have led to the search for alternative agents (Haapasalo et al., 2014). It has previously been shown that the gold standard irrigation protocol (NaOCl followed by EDTA) could not eradicate *E. faecalis* and *C. albicans* biofilms (Alshanta et al., 2020). This was not attributed to the buffering effect of dentine or the complex root canal anatomy, but rather to the high tolerance of biofilm to antimicrobials (Alshanta et al., 2020). It is clearly desirable therefore to identify a chelating agent with enhanced antimicrobial properties.

IP6 is a natural organic acid that is extracted from rice bran (Graf, 1983). IP6 has multiple negative charges with an ability to chelate with multivalent cations (Luttrell, 1993; Torres et al., 2005) and has been proposed as a potential alternative to EDTA (Nassar et al., 2015). IP6 effectively removes the smear layer with minimal adverse effects on osteoblast cells (Nassar et al., 2015). Despite use of IP6 in several medical, dental, nutritional, and industrial applications, there is limited knowledge concerning its antimicrobial effectiveness and mode of action. Elucidating such effects might result in the development of novel IP6 products for a range of applications, including in endodontics (Nassar et al., 2021).

In general, EDTA has little or no antibacterial effect (Torabinejad et al., 2003b). Indeed, it has been suggested that EDTA does not exert an antimicrobial effect *per se*, but rather reduces the amount of microorganisms in root canal dentin *via* removal of the smear layer (Di Lenarda et al., 2000). The results of the present *in vitro* study showed that EDTA did not have bactericidal effect against planktonic cultures except for Gram-negative bacteria. This finding agreed with previously published results, where EDTA affected Gram-negative bacteria by combining with cations of the outer cell membrane, leading to altered permeability and metabolism (Vaara, 1992; George et al., 2009). In contrast, a bactericidal effect on Gram-positive bacteria, specifically *E. faecalis*, has either been absent or minimal (Torabinejad et al., 2003b; Arias-Moliz et al., 2008; Zhang et al., 2015), which is consistent with our present study. It was also reported that different concentrations of EDTA were not able to eradicate *E. faecalis* biofilms (Arias-Moliz et al., 2009) and this is in agreement with our results, where we revealed an inability of EDTA to eradicate established biofilms of *E. faecalis* and all tested organisms except *E. coli*. Moreover, our data showed that EDTA did not have a biocidal effect on *C. albicans* and was unable to eradicate its biofilms. As expected, NaOCl showed biostatic, biocidal and antibiofilm activity against all tested organisms, where the minimum concentration needed for NaOCl to eradicate established bacterial biofilms was the same, or only 2-fold greater than the concentration required to kill planktonic counterparts. However, the minimum NaOCl concentration needed for *C. albicans* biofilms eradication was 8-fold higher than the concentration needed to kill planktonic counterparts and this is indicative of higher tolerance of *C. albicans* biofilms to antimicrobials (Alshanta et al., 2019; Alshanta et al., 2020).

For the first time, the antimicrobial and antibiofilm effects of IP6 using a broad spectrum of microorganisms have been reported. To the best of our knowledge, no study has reported the optimal irrigation time required for different concentrations of IP6 to exert antimicrobial effects against *E. faecalis*. Our findings showed that IP6 had biostatic and biocidal activities against a variety of microbial strains, including Gram-positive and Gram-negative bacteria, drug resistant strains, and *C. albicans*. Furthermore, a bactericidal effect on *E. faecalis*, was achieved after a contact time of 5 min with 0.5%, 1% and 2% IP6. A reduced contact time of 30 s was required for 5% IP6 to be bactericidal. IP6’s rapid action highlights its potentials for use in strategies where rapid bactericidal activity is required. The broad-spectrum antibacterial activity of IP6 and its rapid action, may indicate that its bactericidal mechanism of action is through membrane disruption. Few studies have elucidated the antimicrobial aspects of IP6 and most, if not all, emanate from the food industry where IP6 is tested for its ‘environmentally friendly’ application for food preservation and safety, and prolonging food shelf-life (Kim and Rhee, 2016; Boukhris et al., 2020). Therefore, to aid comparison of our results with others, we included *P. aeruginosa* and *E. coli* in our experiments. Kim and Rhee (2016) showed that IP6 exerted its bactericidal effects at higher concentrations compared to our results, and this may be explained by the different *E. coli* strains used in the studies. Meanwhile, Boukhris et al. (2020) found that IP6 exerted inhibitory effects on *S. aureus*, *E. coli* and *P. aeruginosa* at lower concentrations than those reported in this current study. It has been suggested that IP6 exerts its bactericidal effect on *E. coli* through disruption of the cell membrane (Kim and Rhee, 2016), thus causing excessive cell permeability, which then leads to changes in cell morphology and reduction in intracellular ATP concentration (Zhou et al., 2019). Deferrioxamine, a drug that has shown the ability to disrupt biofilms integrity (Ishida et al., 2011), was found to have proprieties similar to IP6, where both have significant iron-chelating properties (Hawkins et al., 1993). Studies have shown that iron-chelating agents are effective against pathogenic organisms (Coraça-Huber et al., 2018; Scott et al., 2021), presumably because iron is an important component for bacterial proliferation and biofilm formation (Lin et al., 2012; Farrand et al., 2015). Hence, it is plausible to speculate that iron-chelation by IP6 might play a role in its broad-spectrum antibacterial and antibiofilm activity reported in this study. Further *in vitro* and *in vivo* studies are, however, required to fully understand its mechanism.

The failure of root canal treatment is not uncommon and likely due to the high tolerance of associated biofilms to antimicrobials (Ricucci et al., 2013). Therefore, is it imperative for root canal irrigants to eradicate these recalcitrant biofilms. For the first time, IP6 has been shown to be effective in eradicating biofilms of a variety of Gram-positive, Gram-negative bacteria and *C. albicans*. The superiority of IP6 over EDTA in terms of its broad antimicrobial and antibiofilm activity has been demonstrated. The fact that IP6’s antimicrobial activity required minimal time, coupled with previous data showing its ability to remove smear layer while being more biocompatible than EDTA (Nassar et al., 2015), indicates that IP6 is potentially an ideal root canal chelating agent. In this present *in vitro* study, a single-species biofilm model was employed to investigate IP6 antibiofilm activity, however, since most root canal infections are polymicrobial in nature, our future investigations will aim to investigate IP6 activity against complex polymicrobial biofilms. Further *in vitro* and *in vivo* studies are also warranted to determine optimal concentrations for the clinical use of IP6.

## Conclusion

In summary, within the limitation of this *in vitro* study, our data revealed the antimicrobial efficiency of IP6 against planktonic cultures of microorganisms with a rapid bactericidal action against *E. faecalis* planktonic cultures. Furthermore, for the first time we reported the antibiofilm efficacy of IP6 against a range of microorganisms with clinical relevance in dentistry and other human infections.

## Data Availability Statement

The original contributions presented in the study are included in the article. Further inquiries can be directed to the corresponding author.

## Author Contributions

RN, MN, MV, AS and DW conceived the study. RN designed and undertook laboratory experiments, analysed the data and wrote the first draft of the manuscript. FA contributed to data collection. MN, AS and DW contributed to data analysis. RN, MN, MV, NN, EK, AS and DW contributed to critical review and interpretation. All authors contributed to manuscript revision and approved the submitted version.

## Funding

The authors declare the receipt of the following financial support for the research, authorship, and/or publication of this article: This work was supported by funding from the College of Medicine, Mohammed Bin Rashid University of Medicine and Health Sciences (Grant number: MBRU-CM-RG2019-02).

## Conflict of Interest

The authors declare that the research was conducted in the absence of any commercial or financial relationships that could be construed as a potential conflict of interest.

## Publisher’s Note

All claims expressed in this article are solely those of the authors and do not necessarily represent those of their affiliated organizations, or those of the publisher, the editors and the reviewers. Any product that may be evaluated in this article, or claim that may be made by its manufacturer, is not guaranteed or endorsed by the publisher.
